# Regulation of Vascular Calcification by M1-Type Macrophage-Derived Semaphorin 4D

**DOI:** 10.3390/ijms26115071

**Published:** 2025-05-24

**Authors:** Hyun-Joo Park, Yeon Kim, Mi-Kyoung Kim, Hyung Joon Kim, Soo-Kyung Bae, Moon-Kyoung Bae

**Affiliations:** 1 Department of Oral Physiology, School of Dentistry, Pusan National University, Yangsan 50612, Republic of Korea; 2 Dental and Life Science Institute, School of Dentistry, Pusan National University, Yangsan 50612, Republic of Korea; 3 Department of Dental Pharmacology, School of Dentistry, Pusan National University, Yangsan 50612, Republic of Korea

**Keywords:** semaphorin 4D, macrophage, vascular smooth muscle cells, vascular calcification

## Abstract

Vascular calcification is a critical pathological hallmark of cardiovascular diseases. Although previous studies have indicated that M1 macrophages significantly promote calcification, the exact underlying mechanisms remain unclear. This study examined whether semaphorin 4D (Sema4D), a class IV semaphorin involved in atherosclerosis development, is secreted by M1 macrophages and contributes to the calcification of vascular smooth muscle cells (VSMCs). We observed elevated expression and secretion of Sema4D in both M1 and M2 macrophages, with significantly higher levels in M1-polarized cells. M1 macrophages promoted VSMC calcification in both co-culture and conditioned medium systems, as evidenced by increased alkaline phosphatase activity, enhanced calcium deposition, and upregulation of osteogenic markers. Notably, neutralization of Sema4D in M1 conditioned medium using pepinemab, an anti-Sema4D antibody, effectively attenuated VSMC calcification induced by M1 macrophages. Conversely, supplementation of conditioned medium with recombinant Sema4D enhanced calcification and osteogenic signaling in VSMCs, further supporting the pro-calcifying role of Sema4D. Collectively, these findings highlight macrophage-derived Sema4D as a pivotal regulator of vascular calcification and a promising therapeutic target.

## 1. Introduction

Semaphorins denote a large family of proteins characterized by both secreted and membrane-bound molecules with a conserved Sema domain at their carboxyl terminus [1]. They were initially identified in developing nerve tissues as axonal guidance proteins and exhibit diverse roles in the nervous and immune systems [2]. Semaphorin 4D (Sema4D), or CD100, is a transmembrane glycoprotein belonging to the class IV semaphorin family and exists in membrane-bound and soluble forms [3]. Initially characterized as essential for neuronal development, Sema4D has emerged as a multifunctional signaling protein that interacts with its primary receptors, Plexin-B1 and CD72. Through these interactions, Sema4D mediates both localized and systemic signaling cascades that regulate critical physiological and pathological processes, including immune modulation, angiogenesis, tumor progression, and atherosclerosis [4,5].

Vascular calcification, characterized by the pathological deposition of calcium phosphate crystals in blood vessel walls, significantly contributes to cardiovascular morbidity and mortality, particularly in patients with chronic kidney disease and atherosclerosis [6]. Traditionally viewed as a passive degenerative process, it is now recognized as an actively regulated cascade mechanism involving transdifferentiation of vascular smooth muscle cells (VSMCs) into osteoblast-like cells, apoptosis, and matrix vesicle release [7,8]. Emerging evidence suggests a crucial role for macrophage polarization in this process, with the balance between M1 and M2 phenotypes influencing the progression of vascular calcification [9]. Whereas M1 macrophages appear to promote inflammation and exacerbate vascular calcification, M2 macrophages are associated with tissue repair and the inhibition of calcification [10].

We previously reported that Sema4D knockdown in VSMCs slowed phosphate-induced vascular calcification [11]. Sema4D is expressed and released by macrophages under various pathological conditions [12,13,14]. In the tumor microenvironment, it promotes angiogenesis, and under inflammatory conditions, it contributes to cartilage degradation in articular chondrocytes [15,16]. Despite these findings, the role of macrophage-derived Sema4D in vascular calcification, particularly in response to macrophage crosstalk, remains largely unclear.

In this study, we aimed to investigate the role of Sema4D secreted by M1-type macrophages in the vascular calcification of VSMCs and to elucidate the underlying molecular mechanisms involved in this process.

## 2. Results

### 2.1. Sema4D Expression Is Upregulated in Polarized M1 and M2 Macrophages

**Figure 1:**
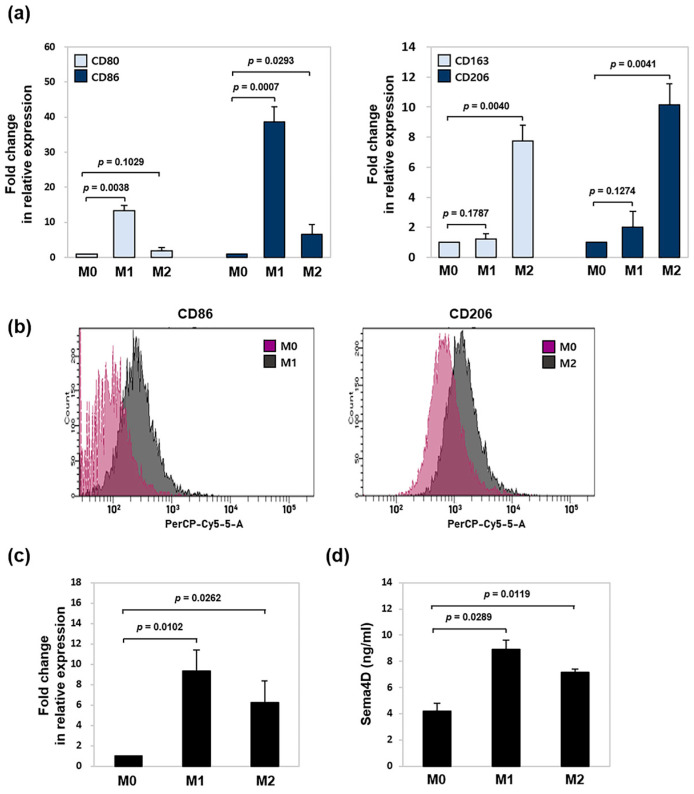
Sema4D expression in M1- or M2-polarized macrophages. BMDMs were cultured in M-CSF-containing growth medium for 7 days (M0 macrophages) and polarized into M1 (100 ng/mL *E. coli* LPS + 20 ng/mL IFN-γ) or M2 (20 ng/mL IL-4 + 20 ng/mL IL-13) macrophages for 24 h. (**a**) Total RNA was isolated and analyzed using RT-qPCR with specific CD80, CD86, CD163, and CD206 primers. Expression in the control (untreated sample) was set to 1, and the values were normalized to β-actin mRNA. (**b**) Cell-surface expression of CD86 and CD206 was measured by flow cytometry. (**c**) Sema4D mRNA was analyzed by RT-qPCR. Expression in the control (untreated sample) was set to 1, and the values were normalized to β-actin mRNA. (**d**) Sema4D secreted into conditioned media by polarized macrophages was measured using ELISA. Data represent the mean ± SD from at least three independent experiments.

M1 and M2 macrophages can be distinguished based on their phenotypic and secretory profiles [17]. To evaluate the expression of Sema4D in distinct macrophage phenotypes, we utilized rat bone marrow-derived macrophages (BMDMs). These cells were first differentiated into M0 macrophages using macrophage colony-stimulating factor (M-CSF), followed by polarization into M1 or M2 subtypes using *Escherichia coli* lipopolysaccharide (LPS) + interferon-gamma (IFN-γ) or interleukin (IL)-4 + IL-13, respectively. Polarization was validated using real-time quantitative PCR (RT-qPCR) of phenotype-specific marker genes. As expected, M1 macrophages exhibited significantly higher CD80 and CD86 expression, whereas M2 macrophages showed marked upregulation of CD163 and CD206 compared to M0 controls (Figure 1a). Consistent with these results, flow cytometry revealed higher surface expression of CD86 in M1 macrophages and CD206 in M2 macrophages (Figure 1b). Next, we assessed Sema4D expression in polarized macrophages. As shown in Figure 1c, Sema4D mRNA levels were elevated in both M1- and M2-polarized macrophages compared to M0 cells, with the highest expression observed in the M1 subtype. The result was confirmed by measuring Sema4D secretion using an enzyme-linked immunosorbent assay (ELISA) analysis (Figure 1d).

### 2.2. M1 Macrophages Promote VSMC Calcification via Co-Culture and Conditioned Medium

**Figure 2:**
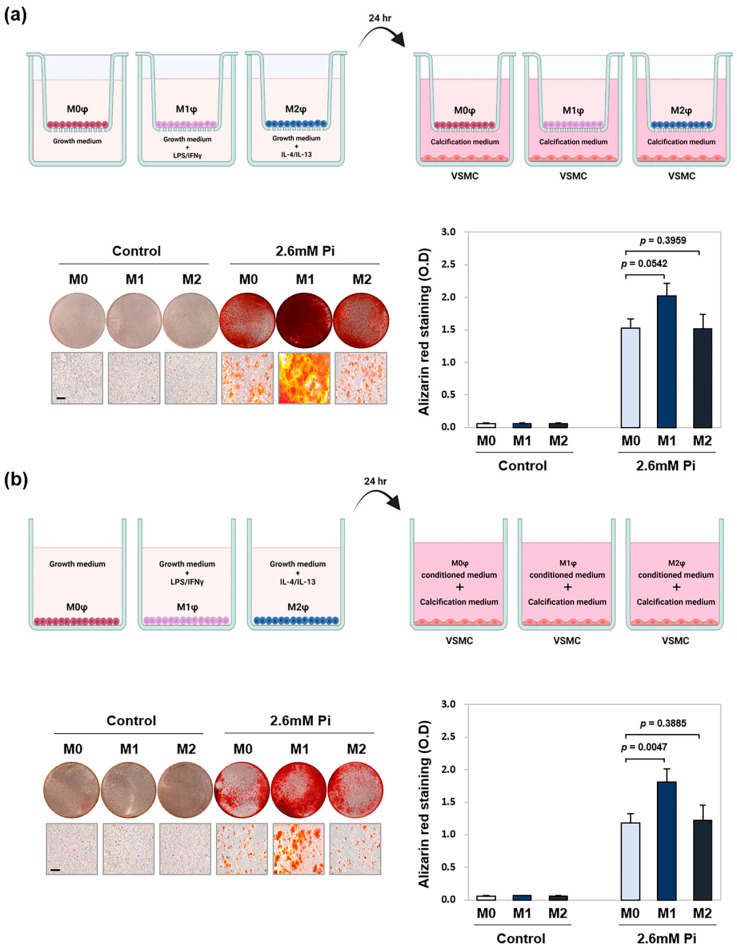
Effect of M1- or M2-polarized macrophages on VSMC calcification. VSMCs were seeded in 24-well plates and co-cultured with M0-, M1- or M2-polarized macrophages placed in Transwell inserts. The co-culture was maintained in a calcification medium (2.6 mM inorganic phosphate (Pi)) for 5 d. (**a**) Calcified nodule formation was assessed by ARS staining, followed by absorbance measurement to quantify the degree of mineralization. Calcium deposits were visualized using a digital camera and microscopy. Scale bae = 50μm. The upper panel shows a schematic illustration of M0, M1, and M2 macrophages co-cultured with VSMCs. (**b**) VSMCs were separately cultured with a 1:1 mixture of calcification medium (2.6 mM Pi) and conditioned medium from M0, M1, or M2 macrophages. The upper panel presents a schematic representation of conditioned media treatment, and the lower panel shows absorbance data quantifying ARS-stained mineral deposition. Data represent the mean ± SD from at least three independent experiments.

To investigate the effect of the macrophage subtype on vascular calcification, we employed a transwell co-culture system in which VSMCs were cultured with polarized macrophages. Macrophages were first polarized for 24 h within Transwell inserts and then co-cultured with VSMCs in a calcification medium for 5 d. As shown in Figure 2a, Alizarin red S (ARS) staining revealed significantly stronger calcification in VSMCs co-cultured with M1 macrophages compared to those with M0 macrophages. In contrast, co-culture with M2 macrophages did not significantly affect calcification levels relative to M0 controls. To determine whether macrophage-secreted factors were responsible for the effect, we treated VSMCs with conditioned media from M0, M1, or M2 macrophages. VSMCs were cultured for 5 d in a 1:1 mixture of calcification medium and conditioned media. Consistent with co-culture results, VSMCs exposed to M1 conditioned medium exhibited significantly stronger calcification compared to treatment with M0 conditioned medium (Figure 2b), while M2 conditioned medium had no significant effect (Figure 2b).

### 2.3. M1 Macrophages Promote Osteogenic Differentiation of VSMCs

**Figure 3:**
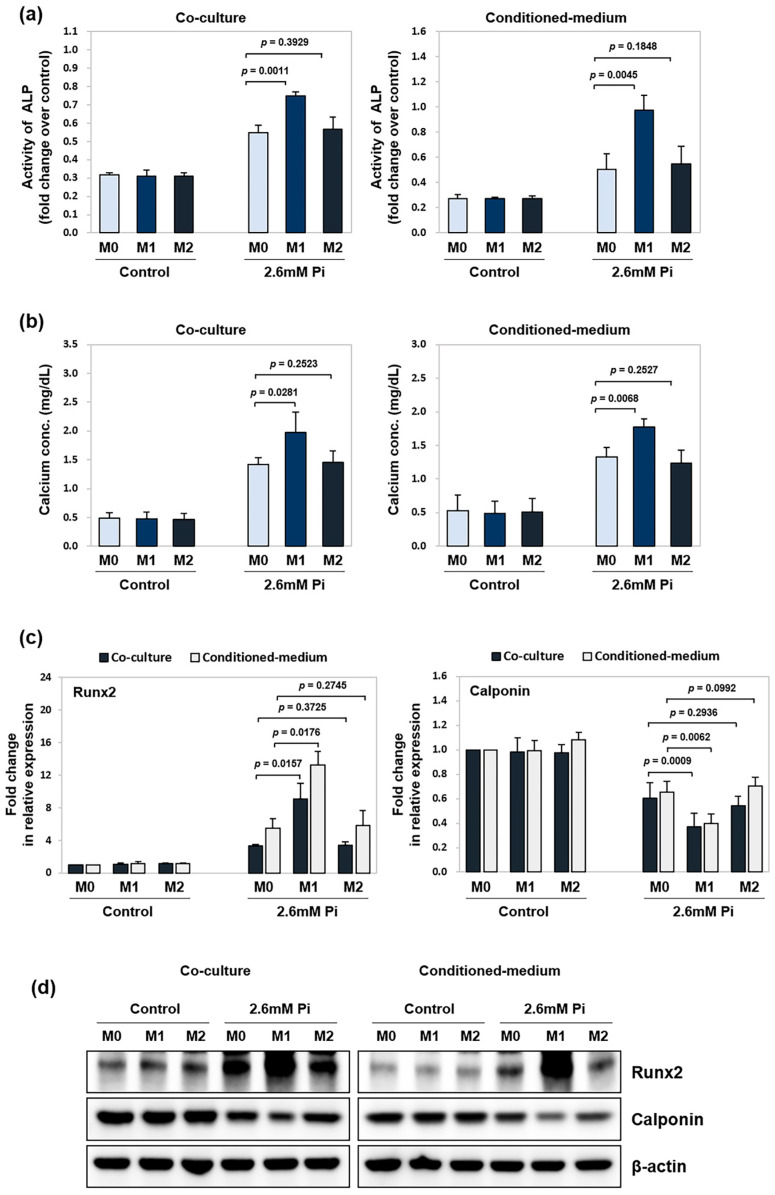
Effect of M1- or M2-polarized macrophages on the expression of calcification-related markers in VSMCs. VSMCs were seeded in 24-well plates and cultured in a calcification medium (2.6 mM Pi) for 5 d. They were then co-cultured with M0-, M1-, or M2-polarized macrophages in Transwell inserts or treated with a 1:1 mixture of calcification medium (2.6 mM Pi) and conditioned medium collected from the respective macrophage types. (**a**) ALP activity was measured and normalized to cellular protein content for quantitative analysis. (**b**) Calcium content was quantified using a colorimetric assay kit. (**c**) The mRNA expression of Runx2 and calponin was analyzed by RT-qPCR. (**d**) The Runx2 and calponin protein levels were analyzed by western blotting and normalized to β-actin. Data represent the mean ± SD from at least three independent experiments.

Alkaline phosphatase (ALP) activity and calcium content are established indicators of VSMC calcification [18]. Using these markers, we examined how different macrophage subtypes influence VSMC calcification through both co-culture and conditioned medium models. As shown in Figure 3a, ALP activity in VSMCs rose significantly in the presence of either co-cultured M1 macrophages or M1 conditioned medium compared to M0 controls. Similarly, calcium content was markedly elevated in the M1 group, whereas no significant difference was observed between M2 and M0 conditions (Figure 3b). To further characterize the molecular changes associated with M1-induced calcification, we examined the expression of osteogenic and contractile markers in VSMCs. As shown in Figure 3c, exposure to M1 cells or their medium upregulated mRNA levels of Runx2 while significantly downregulating the contractile marker calponin expression compared to M0 treatment. Western blot analysis confirmed increased protein levels of Runx2 and a notable reduction in calponin in response to M1 macrophage stimulation (Figure 3d).

### 2.4. Blockade of Sema4D Reduces VSMC Calcification Induced by Exposure to M1 Macrophage-Conditioned Medium

**Figure 4:**
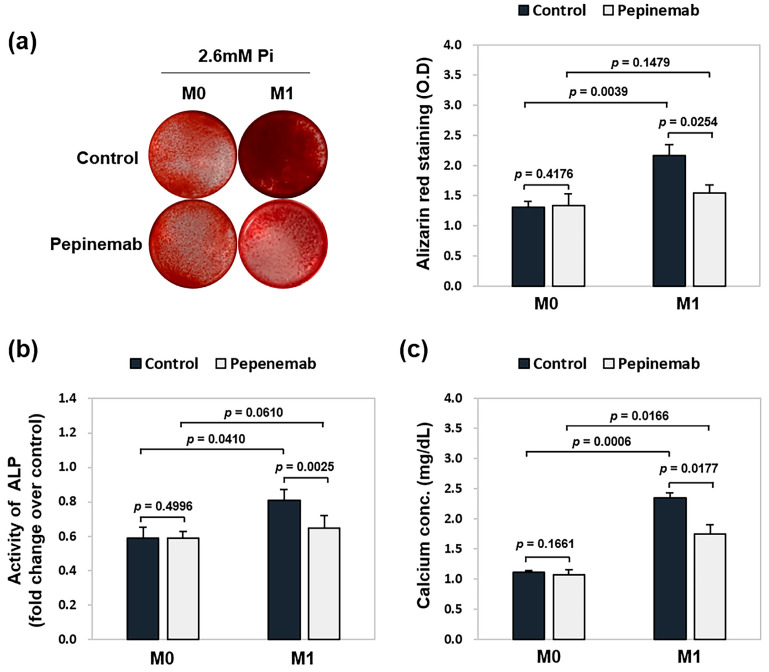

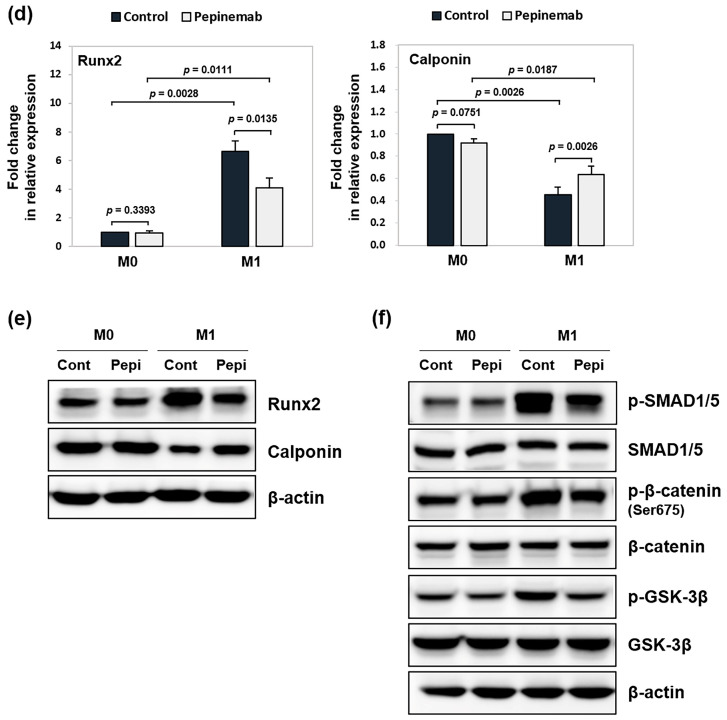
Effect of pepinemab on VSMC calcification induced by M1 conditioned medium. VSMCs were seeded in 24-well plates and cultured for 5 days in a 1:1 mixture of calcification medium (2.6 mM Pi) and conditioned medium collected from M0- or M1-polarized macrophages, with or without pepinemab or its isotype control. (**a**) Calcified nodule formation was assessed by ARS staining, followed by absorbance measurement to quantify the degree of mineralization(**b**) ALP activity was measured and normalized to cellular protein content for quantitative analysis. (**c**) Colorimetric quantification of calcium content using a calcium assay kit. (**d**) The mRNA expression of Runx2 and calponin was analyzed by RT-qPCR. (**e**,**f**) Changes in the levels of Runx2, calponin, phospho-SMAD1/5, SMAD1/5, phospho-β-catenin, β-catenin, phospho-GSK-3β, and GSK-3β were evaluated by western blotting. β-actin was used as a loading control. Data represent the mean ± SD from at least three independent experiments.

We have previously shown that the inhibition of Sema4D in VSMCs attenuated the phosphate-induced osteogenic phenotype of VSMCs [11]. In the present study, we examined whether the pro-calcifying effect of M1 macrophages was regulated through macrophage-derived Sema4D. To address this, VSMCs were cultured in M0- or M1-conditioned media (1:1 mixture with calcification medium) in the presence or absence of pepinemab, a neutralizing antibody against Sema4D. As shown by ARS staining in Figure 4a, calcium deposition induced by M1 conditioned medium decreased significantly upon pepinemab treatment, whereas no effect was observed with M0 conditioned medium. Similarly, pepinemab treatment markedly reduced ALP activity and calcium content in VSMCs exposed to M1-conditioned medium (Figure 4b,c). At the molecular level, RT-qPCR analysis showed that pepinemab reversed M1-induced transcriptional changes, significantly reducing the expression of Runx2 while restoring that of calponin (Figure 4d). Western blot analysis revealed that pepinemab treatment reduced the protein levels of Runx2 while increasing calponin expression (Figure 4e). To further explore the signaling mechanisms involved, we examined SMAD1/5 and β-catenin pathways, both of which contribute to osteogenic transdifferentiation of VSMCs [19,20]. In particular, β-catenin is a central mediator of Wnt signaling, with its activation partially regulated by glycogen synthase kinase-3β (GSK-3β) [21]. As shown in Figure 4e, M1 conditioned medium significantly increased the phosphorylation of SMAD1/5, β-catenin, and GSK-3β compared to M0 conditioned medium. Notably, pepinemab treatment dramatically reduced the phosphorylation of SMAD1/5 and β-catenin, while the phosphorylation of GSK-3β was decreased to levels comparable to those observed under M0 conditions.

### 2.5. Exogenous Sema4D Enhances VSMC Calcification in the Presence of M1-Conditioned Medium

**Figure 5:**
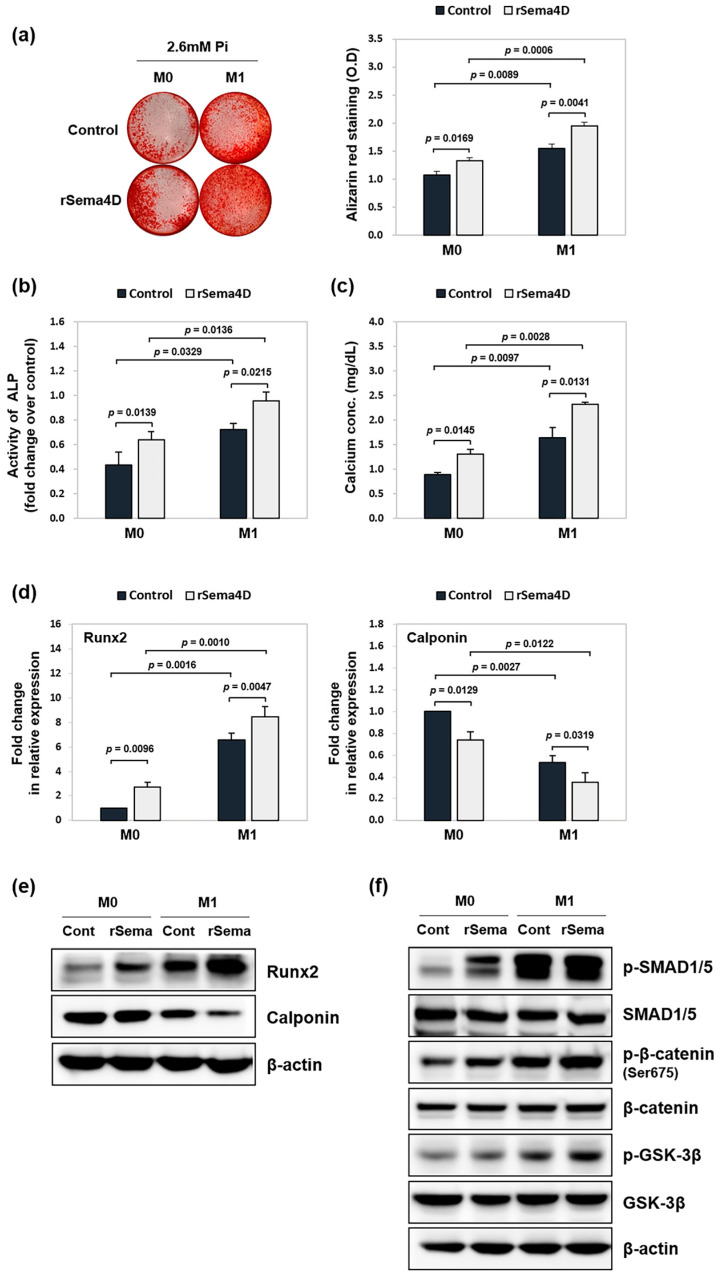
Effect of rSema4D on VSMC calcification induced by M1 conditioned medium. VSMCs were seeded in 24-well plates and cultured in a 1:1 mixture of calcification medium (2.6 mM Pi) and conditioned medium collected from M0- or M1-polarized macrophages. rSema4D (200 ng/mL) was added to the culture medium in selected conditions. After 3 d of culture, the cells were subjected to ARS staining (**a**), ALP activity assay (**b**), and calcium content measurement (**c**). (**d**) The mRNA expression of Runx3 and calponin was examined by RT-qPCR. (**e**,**f**) Protein levels of Runx2, calponin, phospho-SMAD1/5, SMAD1/5, phospho-β-catenin, β-catenin, phospho-GSK-3β, and GSK-3β were assessed by western blotting. β-actin served as a loading control. Data represent the mean ± SD from at least three independent experiments.

As demonstrated above, the neutralization of soluble Sema4D derived from M1 macrophages attenuated VSMC calcification, indicating a functional role for Sema4D in this process. To further validate this finding, we investigated whether exogenous recombinant Sema4D (rSema4D) could enhance calcification under the same conditions. VSMCs were incubated in a 1:1 mixture of calcification medium and conditioned media derived from M0 or M1 macrophages, with or without rSema4D supplementation. As shown in Figure 5a, treatment with rSema4D significantly increased calcium deposition in VSMCs exposed to M1-conditioned medium. Although the M0-conditioned medium alone did not induce calcification, adding rSema4D led to a modest increase in calcium accumulation under these conditions. Consistently, ALP activity and calcium content were further elevated in the rSema4D-treated samples exposed to M1 conditioned medium, and a slight increase was also observed when rSema4D was added to M0 conditioned medium (Figure 5b,c). At the molecular level, rSema4D treatment upregulated the expression of Runx2 and downregulated calponin in VSMCs exposed to either M0 or particularly M1 conditioned medium (Figure 5d,e). At the same time, rSema4D enhanced the phosphorylation of SMAD1/5, β-catenin, and GSK-3β upon exposure to M1 conditioned medium (Figure 5f). A similar but weaker activation pattern was also observed in the M0-conditioned medium treated with rSema4D.

## 3. Discussion

Recent studies have increasingly highlighted the complex role of macrophages in vascular calcification, demonstrating both promoting and inhibiting effects [17,22]. Macrophage polarization significantly influences the progression of vascular calcification, with pro-inflammatory M1 macrophages promoting calcification through IL-1β, IL-6, and tumor necrosis factor-alpha (TNF-α) secretion, while anti-inflammatory M2 macrophages inhibit calcification by increasing extracellular ATP and pyrophosphate levels [23,24,25]. Macrophages secrete several osteogenic proteins, including BMP2, Runx2, and osteopontin, significantly influencing the calcification process [26,27]. BMP2 acts as a crucial regulator by inducing the expression of Msx2 and Runx2 in VSMCs, thereby promoting osteogenic transformation and calcification [28]. Soluble Sema4D secreted from LPS-primed macrophages triggers activation of the inflammatory transcription factor NF-κB, which orchestrates the expression of pro-inflammatory cytokines and elicits pro-inflammatory response in mouse articular chondrocytes [16]. Transfection with Sema4D-specific siRNA partially attenuated the mRNA upregulation and protein secretion of IL-6, TNF-α, and BMP-2 in M1 macrophages (Supplementary Figure S1). These data imply that the calcification of VSMCs is modulated not only directly by Sema4D secreted from M1 macrophages but also indirectly through pro-inflammatory and osteogenic mediators, such as IL-6, TNF-α, and BMP-2, regulated by Sema4D signaling. Further investigations into these regulatory mechanisms are currently underway.

Previous studies have indicated that Sema4D plays an essential regulatory role in macrophage polarization, particularly toward the M2 phenotype under various pathological conditions. Indeed, grafts modified with Sema4D have been shown to accelerate endothelialization and modulate immune responses by shifting macrophage differentiation toward the anti-inflammatory M2 subtype [29,30]. Recently, studies in choroidal neovascularization have revealed that Sema4D knockout markedly inhibits M2 polarization in senescent macrophages, particularly in aged mice [31]. However, the molecular mechanisms through which Sema4D orchestrates macrophage polarization toward M1 or M2 phenotypes, specifically within the vascular calcification microenvironment, remain partly unclear. Further studies are necessary to clarify whether Sema4D influences the polarization state of macrophages, a process associated with vascular calcification.

Sema4D mRNA expression has been regulated by hypoxia-inducible factor-1, microRNA-214 (miR-214), or RNA-binding protein HuR in various cell types [32,33,34]. In contrast, the elevated levels of soluble Sema4D protein in rheumatoid arthritis are not due to increased Sema4D mRNA expression but are driven by a disintegrin and metalloproteinase with thrombospondin motif 4 (ADAMTS4)-mediated proteolytic cleavage of membrane-bound Sema4D [35]. Besides ADAMTS-4, a disintegrin, metalloproteinase domain 17, and membrane-type-1 matrix metalloproteinase have also been identified as sheddases, which cleave membrane-bound Sema4D to generate its soluble form [36]. Notably, ADAMTS-4 expression is induced during monocyte-to-macrophage differentiation and is further enhanced upon stimulation by the inflammatory cytokines IFN-γ and TNF-α. Indeed, ADAMTS-4 is abundant in macrophage-rich regions within human atherosclerotic plaques [37]. Given these findings, we hypothesize that ADAMTS4-mediated Sema4D shedding could potentially be enhanced during differentiation toward the pro-inflammatory M1 macrophage phenotype; however, this remains to be elucidated. Future studies will be needed to investigate both the transcriptional regulation and post-translational shedding mechanisms of Sema4D in this context.

Our findings suggest that macrophage-derived soluble Sema4D contributes to vascular calcification and may serve as a promising biomarker and therapeutic target. This is particularly important given the established association of vascular calcification with high-risk conditions such as diabetes mellitus, chronic kidney disease (CKD), and atherosclerosis [6]. In the context of CKD, where hyperphosphatemia is a key driver of pathological vascular calcification, targeting Sema4D may offer particular clinical benefit. Supporting this, recent studies have shown that members of the semaphorin family, including semaphorin 3A, are associated with kidney injury and disease progression in CKD patients [38]. Furthermore, our previous work demonstrated that inhibition of the Sema4D–Plexin-B1 axis effectively prevents phosphate-induced calcification in VSMCs [11], directly implicating Sema4D in the pathogenesis of vascular calcification under hyperphosphatemic conditions. In addition, Sema4D deficiency reduces kidney injury in glomerulonephritis models, likely by dampening immune responses through its receptors [39]. These findings support the potential of Sema4D as a therapeutic target in CKD-related cardiovascular complications. Recent reviews, including that by Yin et al., have highlighted the critical role of semaphorin signaling in vascular pathology, further supporting the clinical significance of our findings [40,41]. Notably, increased levels of circulating Sema4D have been observed in patients with heart failure, strongly supporting its feasibility as a clinical biomarker [42]. To validate the physiological relevance of our in vitro observations, future studies will incorporate in vivo models—such as Sema4D-deficient mice or experimental calcification systems—as well as analyses of human patient samples to assess its diagnostic and therapeutic potential.

In conclusion, our study identifies macrophage-derived Sema4D as a critical positive regulator of vascular calcification, which acts by activating osteogenic pathways in VSMCs. Sema4D offers a novel and promising target to mitigate calcification in cardiovascular diseases. Further investigations into Sema4D signaling may provide significant insights into new preventive and therapeutic strategies for vascular calcification.

## 4. Materials and Methods

### 4.1. Reagents and Antibodies

Recombinant Sema4D and anti-Sema4D antibodies were purchased from BD Biosciences (San Jose, CA, USA). Pepinemab was obtained from MedChemExpress (Monmouth Junction, NJ, USA). PerCP/Cy5.5-conjugated anti-CD86 and anti-CD206 antibodies were purchased from BioLegend (San Diego, CA, USA). Antibodies against phospho-SMAD1/5, phospho-β-catenin, β-catenin, phospho-GSK-3β, GSK-3β, and Runx2 were obtained from Cell Signaling Technology (Danvers, MA, USA). Anti-calponin antibody was obtained from Abcam (Cambridge, UK), and an anti-β-actin antibody was purchased from Bioworld Technology (St. Louis Park, MN, USA). Anti-SMAD1/5 and the IgG4 isotype control antibodies were both obtained from Invitrogen (Carlsbad, CA, USA). Horseradish peroxidase-conjugated goat anti-mouse and anti-rabbit IgG secondary antibodies were purchased from Thermo Fisher Scientific (Waltham, MA, USA).

### 4.2. Cell Culture and Polarization

VSMCs were isolated from the thoracic aortas of 3-week-old male Sprague–Dawley rats (Samtaco, Seoul, Republic of Korea) and cultured in Dulbecco’s Modified Eagle’s Medium (DMEM) supplemented with 10% fetal bovine serum (FBS) and 1% penicillin–streptomycin (all Thermo Fisher Scientific) [43]. Cells were maintained at 37 °C in a humidified atmosphere containing 95% air and 5% CO_2_. BMDMs were cultured and identified as described previously [44]. Briefly, rat bone marrow cells were cultured in DMEM supplemented with 30 ng/mL macrophage colony-stimulating factor (M-CSF, PeproTech, Rocky Hill, NJ, USA), 10% FBS, 1% penicillin–streptomycin, and 5 μg/mL Plasmocin^®^ (Invitrogen) for 7 days. These cells were defined as M0 macrophages. For M1 polarization, M0 macrophages were stimulated with 100 ng/mL LPS (InvivoGen, San Diego, CA, USA) and 20 ng/mL IFN-γ (R&D Systems, Minneapolis, MN, USA) for 24 h. For M2 polarization, M0 macrophages were treated with 20 ng/mL IL-4 and IL-13 (both from R&D Systems) for 24 h [45]. All cells were cultured at 37 °C in a humidified environment containing 95% air and 5% CO_2_.

### 4.3. Induction and Quantification of Calcification

To induce calcification, a solution of Pi containing Na_2_HPO_4_ and NaH_2_PO_4_ (pH 7.4), was added to serum-supplemented DMEM at a final concentration of 2.6 mM. This Pi-containing medium was subsequently used as the calcification medium for both the co-culture and conditioned media experiments. Following the indicated incubation periods, cellular calcium content and ALP activity were assessed using a Calcium Colorimetric Assay Kit (BioVision, Milpitas, CA, USA) and an ALP Assay Kit (Takara Bio Inc., Shiga, Japan), respectively.

### 4.4. Co-Culture of VSMCs and Macrophage Subtypes in a Transwell System

M0 macrophages were seeded into the upper inserts of Transwell plates (Corning, Corning, NY, USA) and subsequently polarized into M1 or M2 phenotypes as previously described. One day before polarization was completed, rat VSMCs were seeded into the lower wells of a separate 24-well plate and allowed to adhere overnight. After polarization, the Transwell inserts were gently washed with phosphate-buffered saline (PBS) and placed onto the VSMC-containing wells. Co-cultures were maintained in calcification medium (2.6 mM Pi) for 5 days. The medium and transwell inserts containing polarized M0, M1, or M2 macrophages were replaced every other day.

### 4.5. Culture of VSMCs with Conditioned Medium from Polarized Macrophages

Rat VSMCs were seeded into 24-well plates and allowed to adhere overnight. The culture medium was then replaced with a 1:1 mixture of macrophage-conditioned media (from M0, M1, or M2 macrophages) and calcification medium (2.6 mM Pi). VSMCs were cultured under these conditions for 5 days. To prepare the conditioned media, M0 macrophages were seeded into separate plates and polarized into M1 or M2 phenotypes for 24 h as described above. Following polarization, cells were gently washed with PBS and cultured for an additional 24 h in serum-free medium. The resulting supernatants were collected, centrifuged to remove debris, and used as macrophage-conditioned medium.

### 4.6. ARS Staining and Quantification

Cells cultured on plastic supports were fixed with 4% paraformaldehyde for 15 min and stained with 1 mg/mL ARS (Sigma-Aldrich, St. Louis, MO, USA) solution for 30 min at 37 °C. After staining, samples were rinsed with distilled water to remove excess dye, and calcium deposits were visualized and photographed under a light microscope. Prior to quantification, the stained calcium deposits were destained with 10% acetic acid, and the resulting solution was transferred to a 96-well plate. Absorbance was measured at 420 nm using a multi-detection microplate reader (Dynex Technologies, Chantilly, VA, USA) to assess the extent of calcification.

### 4.7. Quantitative Real-Time Reverse-Transcription PCR

Total RNA was isolated using a RiboEx kit (GeneAll, Seoul, Republic of Korea) and reverse transcribed using a reverse transcription kit (Promega, Madison, WI, USA), followed by RT-qPCR using the SYBR Green premix (Enzynomix, Daejeon, Republic of Korea). Cycling parameters included one cycle at 95 °C for 10 min, followed by amplification for 40 cycles at 95 °C for 15 s, 60 °C for 60 s, and 72 °C for 7 s. The process was performed using a CFXDuet Real-Time PCR Detection System (Bio-Rad, Hercules, CA, USA). The primers used were as follows: β-actin 5′-AGGGAAATCGTGCGTGAC-3′ and 5′-CGCTCATTGCCGATAGTG-3′; CD80 5′-AGTTGCCAGCTGATGCAGGA-3′ and 5′-ACCAGCAGCAGCACACAGAG-3′; CD86 5′-TCGGTGTTGGCCTATCTGCT-3′ and 5′-ACTACGAGCTCACTCGGGCTT-3′; CD163 5′-CCGTGACGCTTCTGTGGTGT-3′ and 5′-TGGTCCGGATCCCTCACTGG-3′; CD206 5′-AGCCTACACCCGCTCCTCAA-3′ and 5′-GCGCGTTGTCCATGGTTTCC-3′; Sema4D 5′-TGGGAGCACGGAGAGGTAGG-3′ and 5′-CTTCCCGGGCACCCACATAC-3′; ALP 5′-TGCTTTGTGTGTGCTGACTGTA-3′ and 5′-AGTGACGGTGTCGTAGCCTTCT-3′; Runx2 5′-GCCGGGAATGATGAGAACTA-3′ and 5′-TGGGGAGGATTTGTGAAGAC-3′; calponin 5′-GAACAAGCTGGCCCAGAAAT-3′ and 5′-GGCCATCCATGAAGTTGCTC-3′.

### 4.8. Western Immunoblot Analysis

Approximately 30 μg of total protein per lane was loaded on 10% bis-acrylamide gels, and electrophoresis was performed at 80 V for 2 h. The separated proteins were transferred to nitrocellulose membranes (Amersham Pharmacia Biotech, Uppsala, Sweden); the membranes were blocked in 5% skim milk for 1 h under agitation and probed with primary antibodies for 16 h at 4 °C. Then, the membranes were washed three times for 10 min each with PBS and incubated for 1 h at room temperature with horseradish peroxidase-conjugated goat-anti-rabbit IgG or goat-anti-mouse IgG as the secondary antibodies. The signal was developed using an enhanced chemiluminescence solution (Amersham Pharmacia Biotech) and visualized on an Azure 300 Imaging system (Azure Biosystems, Dublin, CA, USA).

### 4.9. Flow Cytometry Analysis

Polarized M1 and M2 macrophages were incubated with PerCP/Cy5.5-conjugated anti-CD86 and anti-CD206 antibodies, respectively, for 30 min at 4 °C in the dark. After incubation, cells were washed twice with PBS containing 1% bovine serum albumin and resuspended in PBS for analysis. Flow cytometry was performed using a BD FACS Canto II flow cytometer (BD Biosciences), and data were analyzed using FlowJo software (version 10.10, Tree Star, Ashland, OR, USA). Unstained and isotype control samples were used to set gates and confirm specificity.

### 4.10. ELISA

The concentration of secreted Sema4D protein in conditioned medium was quantified using an ELISA kit (MyBioSource, San Diego, CA, USA), following the manufacturer’s instructions. Absorbance was measured at 450 nm using a microplate reader (Dynex Technologies). Sema4D levels were calculated by interpolating the absorbance values onto a standard curve generated according to the manufacturer’s protocol.

### 4.11. Statistical Analysis

All data are presented as the mean ± standard deviation (SD) from at least three independent experiments. Statistical significance was assessed using a one-way analysis of variance, followed by Tukey’s honest significant difference post hoc test or Student’s *t*-test, as appropriate. Analyses were performed using IBM SPSS, version 27 (IBM Corp., Armonk, NY, USA). A *p*-value < 0.05 was considered statistically significant.

## Data Availability

The data presented in this study are available upon request from the corresponding author.

## Supplementary Materials

The following supporting information can be downloaded at: https://www.mdpi.com/article/10.3390/ijms26115071/s1.

## Abbreviations

The following abbreviations are used in this manuscript:

VSMCs	Vascular smooth muscle cells
Sema4D	Semaphorin 4D
LPS	Lipopolysaccharide
IFN-γ	Interferon-gamma
IL	Interleukin
ARS	Alizarin red S
ALP	Alkaline phosphatase
GSK-3β	Glycogen synthase kinase-3β
rSema4D	Recombinant semaphorin 4D
TNF-α	Tumor necrosis factor-alpha
BMP-2	Bone morphogenic protein 2
ADAMTS-4	Metalloproteinase with thrombospondin motif 4
DMEM	Dulbecco’s Modified Eagle’s Medium
FBS	Fetal bovine serum
PBS	Phosphate-buffered saline
ELISA	Enzyme-linked immunosorbent assay
Pi	Inorganic phosphate
