# Supplementary material for: Regulation of Vascular Calcification by M1-Type Macrophage-Derived Semaphorin 4D
Source: Int J Mol Sci. 2025 May 24;26(11):5071. doi: 10.3390/ijms26115071 (PMC12154369; doi:10.3390/ijms26115071)
Supplement: Supplementary file 1 [file ijms-26-05071-s001.zip › ijms-3641379-supplementary.pdf]

Regulation of vascular calcification by M1-type macrophage-derived semaphorin 4D

Hyun-Joo Park[1,2], Yeon Kim[1,2], Mi-Kyoung Kim[1], Hyung Joon Kim[1,2], Soo-Kyung Bae[2,3], and Moon-Kyoung Bae[1,2]

[1.] Department of Oral Physiology, School of Dentistry, Pusan National University, Yangsan 50612, South Korea; phj3421@hanmail.net (H.J.P.), eenga@naver.com (M.K.K.), graceyeon88@gmail.com (Y.K.), hjoonkim@pusan.ac.kr (H.J.K.)

[2] Dental and Life Science Institute, School of Dentistry, Pusan National University, Yangsan 50612, South Korea; skbae@pusan.ac.kr (S.K.B.)

[3] Department of Dental Pharmacology, School of Dentistry, Pusan National University, Yangsan 50612, South Korea

Supplementary Data

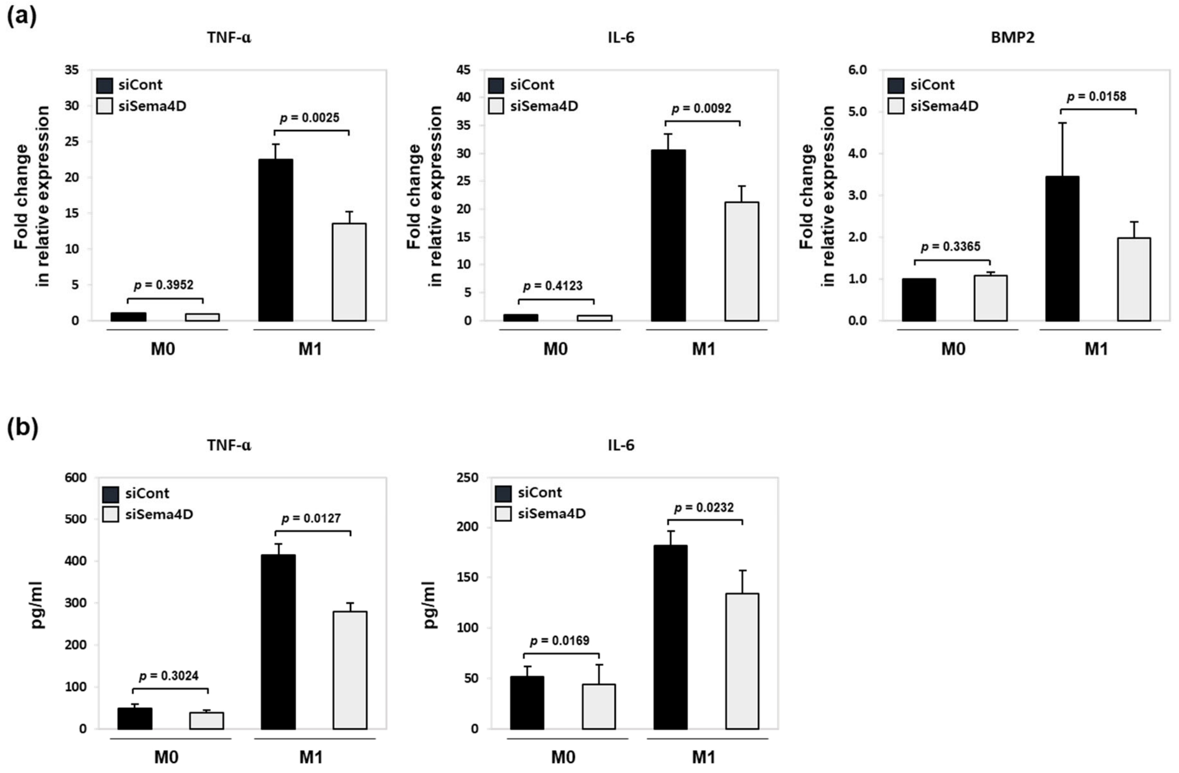

Figure S1: Effect of Sema4D knockdown on the mRNA expression and protein secretion of TNF-α, IL-6 and BMP-2 in M1 macrophages. M0 macrophages were transfected with Sema4D siRNA or negative control siRNA for 24 hours, followed by polarization in medium containing 100 ng/mL *E. coli* LPS and 25 ng/mL IFN-γ for an additional 24 hours. (a) Total RNA was isolated and analyzed using RT-qPCR with specific TNF-α, IL-6 and BMP-2 primers. Expression in the control (untreated sample) was set to 1, and the values were normalized to β-actin mRNA. (b) TNF-α and IL-6 concentration in the cell culture medium was examined by ELISA. Data shown are the mean ± SD, obtained from at least three independent experiments.
